# CXCL12-Abundant Reticular (CAR) Cells Direct Megakaryocyte Protrusions across the Bone Marrow Sinusoid Wall

**DOI:** 10.3390/cells10040722

**Published:** 2021-03-24

**Authors:** Nicole Wagner, Kristina Mott, Berin Upcin, David Stegner, Harald Schulze, Süleyman Ergün

**Affiliations:** 1 Institute of Anatomy and Cell Biology, Julius-Maximilians-University Würzburg, 97070 Würzburg, Germany; nicole.wagner@uni-wuerzburg.de (N.W.); berin.upcin@uni-wuerzburg.de (B.U.); 2 Institute of Experimental Biomedicine, Chair I, University Hospital Würzburg, 97080 Würzburg, Germany; faber_k1@ukw.de (K.M.); stegner@virchow.uni-wuerzburg.de (D.S.)

**Keywords:** megakaryocytes, microvasculature, CXCL12-abundant reticular (CAR)-cells

## Abstract

Megakaryocytes (MKs) release platelets into the lumen of bone marrow (BM) sinusoids while remaining to reside within the BM. The morphogenetic events of this complex process are still not fully understood. We combined confocal laser scanning microscopy with transmission and serial block-face scanning electron microscopy followed by 3D-reconstruction on mouse BM tissue sections. These analyses revealed that MKs in close vicinity to BM sinusoid (BMS) wall first induce the lateral retraction of CXCL12-abundant reticular (CAR) cells (CAR), followed by basal lamina (BL) degradation enabling direct MK-sinusoidal endothelial cells (SECs) interaction. Subsequently, an endothelial engulfment starts that contains a large MK protrusion. Then, MK protrusions penetrate the SEC, transmigrate into the BMS lumen and form proplatelets that are in direct contact to the SEC surface. Furthermore, such processes are induced on several sites, as observed by 3D reconstructions. Our data demonstrate that MKs in interaction with CAR-cells actively induce BMS wall alterations, including CAR-cell retraction, BL degradation, and SEC engulfment containing a large MK protrusion. This results in SEC penetration enabling the migration of MK protrusion into the BMS lumen where proplatelets that are adherent to the luminal SEC surface are formed and contribute to platelet release into the blood circulation.

## 1. Introduction

The transition of a megakaryocyte (MK) into proplatelets and platelets in vitro has been deciphered and studied extensively during the last two decades since the seminal report by Italiano et al. [1] In vivo, however, several additional aspects of platelet biogenesis are required: (i) The MK has to be located at the vessel wall and needs to polarize to avoid ectopic platelet release into the bone marrow (BM) cavity; (ii) the MKs are in steady contact to components of the extracellular matrix (ECM), including collagen, which are strong platelet agonists; (iii) proplatelet protrusions need to cross the endothelium that constitutes the blood-BM barrier. This process has been studied far less and findings remain often inconclusive and partially contradictory, partially due to the limited spatial and temporal imaging of proplatelet transition in vivo.

De Bruyn et al. found in BM of guinea pigs that the sinusoidal walls were devoid of apertures with a continuous endothelial lining, comprising the central, collecting, and primary sinusoids [2]. Nevertheless, the steady egress of mature blood cells from the BM into the blood has consolidated the thinking in the field that the BM endothelial barrier is discontinuous or even fenestrated [3].

Diverse microscopic approaches provided a distinct concept of platelet release into the BM sinusoids (BMS). MKs juxtaposed to sinusoids start to polarize and build cellular protrusions that cross the endothelial barrier where platelet precursors finally shed into the blood stream [4]. Additional concepts suggest that MKs fragment into the blood stream under inflammatory conditions [5] or that sessile MKs contribute to platelet formation by a continuous supply like a “dripping faucet” [6]. Brown et al. showed that protruding MKs lose their organelle-free or marginal zone (MZ) and have anchoring points at the sinusoid wall during the passage. [7] Eckly et al. demonstrated that podosome-like structures contribute to the MK transendothelial passage through newly formed endothelial pores [8].

However, both reports limit to the MK-endothelial interaction and omit further components of the sinus vessel wall, including the basal lamina (BL) and the reticular cells (RCs) of which thin cellular processes are tightly associated with the BL of the sinusoid wall and thus, enwrap most of the sinusoid endothelial cells (SECs). RCs are nestin-positive BM mesenchymal cells, mediating functions otherwise attributed to pericytes. Among the distinct RCs, those expressing high levels of the chemokine CXCL12 have been shown to support the maintenance of hematopoietic stem cells, and are designated CXCL12-abundant reticular (CAR) cells [9]. Recently, Gomariz et al. demonstrated that the murine microvascular system in the femur is covered by CAR-cells, arguing that a potential niche function is not restricted to rare sites within the BM [10]. In this study, we take advantage of complementing confocal laser scanning microscopy with transmission-(TEM) and serial block face scanning-electron microscopy (SBF-SEM) providing evidence that CAR cells are important modulators of steady-state thrombopoiesis.

## 2. Materials and Methods

### 2.1. Mouse Lines

Wildtype C57BL/6 mice, CD41^wt/eYFP^ [11] and Cxcl12^DsRed/+^ mice [12] were used as described. Animal husbandry was performed according to institutional guidelines and approved by the local authorities in the Regierung von Unterfranken (AZ 2-436).

### 2.2. Femur Sectioning and Staining

Mice were sacrificed by cervical dislocation and femurs dissected for fixation in PFA. Fixed femurs were then sliced into 7μm sections on Kawamoto adhesive film and attached to superfrost glass slides as described previously [13]. For immunofluorescence staining sections were thawed and rehydrated in PBS. After blocking with 5% FCS/0.1% Tween-20 sections were probed with primary antibodies against CD105 (eBioscience, San Diego, CA, USA), Endomucin (Santa Cruz, Dallas, TX, USA), laminin α5 (clone 504) [14] collagen type I (ab21286) and β_1_-tubulin (Italiano laboratory, Boston, MA, USA). Corresponding secondary antibodies conjugated with Alexa Fluor 546 (A-110819) or Alexa Fluor 647 (A-21244, A-21247) (all Invitrogen, Carlsbad, CA, USA) were used to detect IgG of rat, rabbit, or mouse origin. Megakaryocytes were stained with an Alexa Fluor 488-coupled antibody raised against GPIX (clone 56F8, Nieswandt laboratory, Würzburg, Germany). For detection of F-actin Alexa Fluor 647-conjugated phalloidin (Invitrogen) was used. All slides were mounted with DAPI-containing medium (Sigma-Aldrich, St. Louis, MO, USA) to visualize nuclei.

### 2.3. Confocal Laser Scanning Microscopy

Immunofluorescence images were taken using a confocal laser scanning microscope (TCS SP8, Leica Microsystems CMS, Wetzlar, Germany) with a 40×/NA 1.3 oil or 100×/NA 1.4 oil objective and LAS X software for image documentation and analysis. Deconvolution of image data and image processing was performed with Huygens essential software (Version 15.10, Scientific Volume Imaging B.V., Hilversum, Netherlands) and ImageJ (Version xx, NIH, MD, USA) software.

### 2.4. Electron Microscopy

TEM sections were analyzed with a LEO912AB transmission electron microscope and SBF-SEM specimens using a Sigma300VP scanning electron microscope (Carl Zeiss Microscopy GmbH, Oberkochen, Germany) equipped with the Gatan3View system (Gatan Inc). Segmentation was performed using the TrakEM2 plug-in [14] of Fiji [15]. 3D rendering model was created using tomviz (https://tomviz.org/, Tomviz Version 1.8.0 accessed on 10 February 2021).

Further detailed description of specimen preparation and image acquisition for electron microscopy are provided in the Supplementary material.

### 2.5. Quantification of MK Protrusions

We used seven whole femur scans for quantification of total MK number (3679) and of proplatelet-forming MKs (54). For representative IF images three femur sections of at least two mice were used for staining. For TEM analysis, 32 individual megakaryocytes (single sections) and 13 megakaryocytes (up to 20 consecutive sections for each megakaryocyte) have been analyzed. From the consecutive sections, five small datasets (ranging from seven to 16 sections) were segmented and reconstructed for the initial analysis of MK transmigration. For SBF-SEM, three datasets of 131 sections and four datasets of 168 sections were analyzed, of which two representative datasets were chosen for segmentation and reconstruction.

## 3. Results

### 3.1. The Megakaryocyte Demarcation Membrane System during Transendothelial Passage

The transendothelial passage of MK cytoplasm requires the supply of membranes, provided by the demarcation membrane sytem (DMS), and the motoric driving force, mediated by microtubules and the actin-myosin-complex. The DMS is a membrane system in the MK center, distinct from the plasma membrane. CD41^wt/eYFP^ mice are reported to constitute a bona fide marker system, leaving the MK plasma membrane virtually unstained. The von Willebrand receptor complex GPIb/IX/V and the phospholipid PI(4,5)P_2_ (stained using the eGFP-tagged PH domain from PLCγ1) have also been used as DMS surrogate markers [15]. Of note, GPIX (CD42a) is also found on the MK surface.

**Figure 1:**
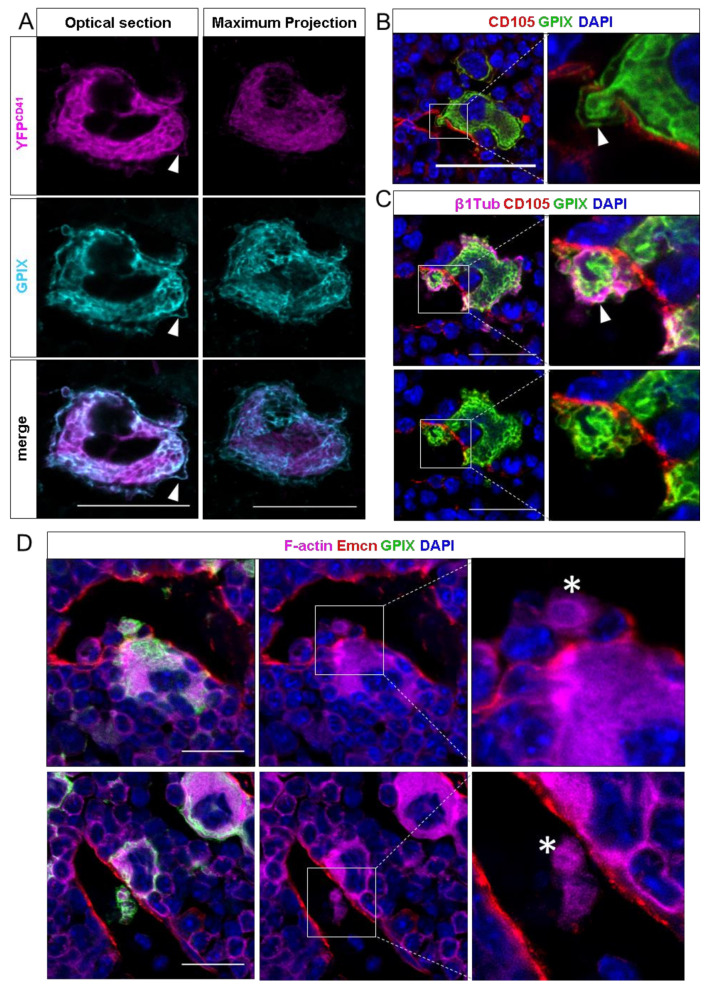
Confocal images of murine femur sections. (**A**) CD41^wt/eYFP^ MKs were co-stained with GPIX. Optical sections show a strong signal overlap for the DMS, but less for the plasma membrane. In maximum projections, the plasma membrane staining becomes less visible. (**B**) Detection of intravasating megakaryocytes by immunohistochemical staining against GPIX and the endothelial marker endoglin (CD105). MKs enter the sinusoidal lumen as large protrusions and maintain a marginal zone (MZ) while crossing the endothelial lining. (**C**) Co-staining of vessels with GPIX and β_1_-tubulin. The MK-specific microtubule isoforms fill the MZ, while protruding the endothelial lining of the sinusoid. (**D**) Visualization of the F-actin cytoskeleton with sinusoids, stained by an additional vessel-marker endomucin (Emcn) and MKs. Large MK protrusions in the vessel lumen are highly actin-enriched, displaying the involvement of the actin-cytoskeleton in the transendothelial passage of MK protrusions. White arrows indicate the MZ, actin-rich proplatelets are marked with asteriscs. Scale bars 20 μm.

To define which membranes are involved during MK transendothelial passage, we used CD41^wt/eYFP^ mice and performed femur sections for confocal laser-scanning microscopy as described recently [13]. First, we performed co-stainings for GPIX with eYFP and—as expected—found a substantial overlap of the two signals, which was much more apparent for internal membranes rather than for the plasma membrane (Figure 1A). In contrast to recent findings [7], we found that most MKs maintain their MZ during the transendothelial passage (Figure 1B). Anti-β_1_-tubulin staining revealed the presence of microtubule filaments at the MK periphery (Figure 1C), potentially providing the cytoskeletal force to bring proplatelets through the endothelial barrier, in concert with actin-based structures (Figure 1D) such as podosomes, [8] although it is known that proplatelets can still be formed without podosomes. [16]

Next, we were interested in the visualization of reticular cells that cover BM SECs. For this, we took advantage of CXCL12-dsRed reporter mice, in which the fluorescent protein dsRed is expressed under control of the CXCL12-promoter. We found that CD105^+^ SECs were broadly covered with CAR-cells and that both MKs in the BM cavity, as well as resting MKs residing at BM vessels were in close contact to CAR-cells (Figure S1).

**Figure 2:**
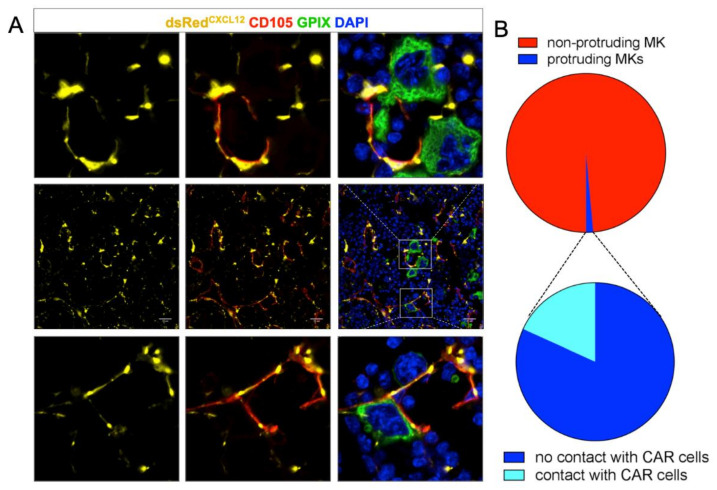
(**A**) dsRed staining of mouse femur sections shows a reticulated pattern (middle row). Resting MKs are in contact with CAR cells (bottom row); the endothelial lining shows pores only when MKs protrude. In these cases, CAR cells were absent or had retracted. (**B**) Quantification of MK-CAR cell contact. About 2% of MKs are protruding the vessel wall; only 25% of them are somewhat in contact with the dsRed expressing CAR cell. Scale bars 20 μm.

In areas of MK penetration, CAR-cells appeared retracted, while the SECs became interrupted (Figure 2A). This is in agreement with findings by Campbell et al., suggesting that the pores are formed either at sites devoid of adventitial cells or that RCs retract. [17] We quantified 3679 MKs from seven complete femur sections and found that less than 25% of vessel-protruding MKs were somewhat in contact with CAR cells (Figure 2B).

### 3.2. Ultrastructure of Megakaryocyte Protrusions during the Transendothelial Passage

**Figure 3:**
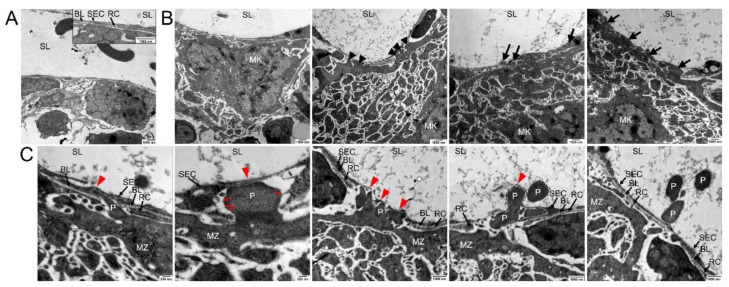
Transmission electron microscopical analysis of megakaryocyte (MK) transmural passage. (**A**) TEM image of components of the blood-BM-barrier, consisting of sinusoidal endothelial cells (SE) which are partially underlined by a basal lamina (BL) and covered by reticular cells (RC) to a large extent. No detection of any preformed pores in the sinusoidal endothelial cells was observed. Sinusoidal lumen (SL). (**B**) TEM images of approaching MKs. Before platelet protrusion, MKs approach the continuous endothelium with their MZ at various points (arrowheads) followed by invagination of MK protrusions into the continuous sinus endothelium (arrows). (**C**) TEM images of different steps of platelet (P) protrusion at higher magnification. At MK/SE contact sites (red arrowhead), MK protrusions provoke local retraction of reticular cells and disintegration of the basal lamina, followed by invagination of MK protrusions into the continuous sinus endothelium, massive stretching of SEs and subsequently rupture of the sinus endothelium and transcellular pore formation. Fine filaments are visible that anchor the protrusion to the SEC (red arrows).

**Figure 4:**
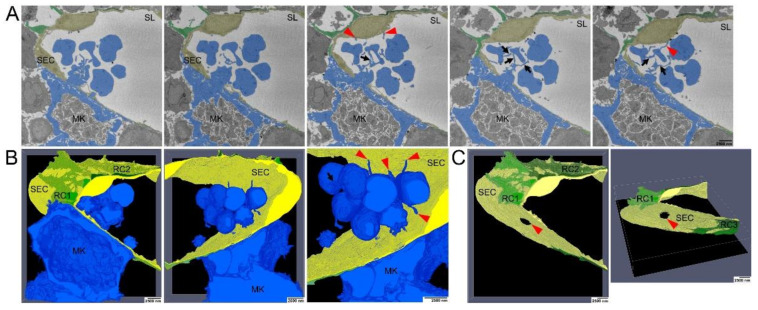
Serial block face scanning electron microscopical analysis of MK transmural passage (**A**) Selected images from single SBF-SEM slices showing a megakaryocyte (MK) (blue) adjacent to the sinusoidal endothelial cells (SECs) (yellow) and (pro)platelets that are still interconnected via small cellular bridges (black arrows) and in contact with SECs (red arrowhead). MK protrusions provoke local retraction of reticular cells (RCs) (green). (**B**) Large-volume reconstruction of the SBF-SEM slices presented in (**A**) showing the relationship between a MK protrusion (blue) and the SECs (yellow). Before release of mature platelets, (pro)platelets are still interconnected via small cellular bridges (black arrow) and they stay still in contact with SECs (red arrowheads). MK protrusions provoke local retraction of RCs (green). (**C**) Large-volume reconstruction of the SBF-SEM slices presented in (**A**) clearly demonstrates MK extensions to cross the blood-BM-barrier by trans-endothelial passage. Model of sinusoidal endothelial cells (yellow) and RCs (green) depicting the site of trans-endothelial passage (hole, read arrowhead) of a MK protrusion (blue) and the local retraction of RCs (green). SBF-SEM dataset: Reconstruction of a 5 µm volume: 168 sections, 10 nm × 10 nm × 30 nm voxel size. Bone marrow of the diaphysis region obtained from murine ulnae. Source data are available for this figure.

As the resolution of confocal microscopy is limited, we performed TEM (Figure 3) and SBF-SEM (Figure 4) and found the BL being present between the adjacent SECs and RCs, but also in RC-free areas of the BMS wall (Figure 3A). We neither detect pre-formed pores nor inter-endothelial gaps with or without close vicinity of MKs to BMS, suggesting a dynamic MK-SEC interaction during the transendothelial passage.

At sites of non-protrusion, the MK MZ is in close vicinity to the BMS, but still separated from the SECs by the BL and RC extensions, thus prohibiting a direct MK-SEC contact (Figure 3B). At areas of direct MK-BMS wall contact, RC processes were absent, probably due to their lateral retraction in response to approaching MKs, and were mostly found positioned at both sides of the MK process (Figure S3). The BL was partially absent and/or degraded (Figure 3B) enabling the direct MK-SEC contact at multiple sites (Figure 3B, black/red arrows). Extended large MK protrusions into the BMS lumen that are initially covered by a thin part of SECs result finally in the SEC penetration and thus subsequently in transmigration of these large MK protrusions into the BMS lumen (Figure 3B, red arrowheads). Sections “en face” of SECs often show cytoplasm of the MK protrusion(s) being completely surrounded by SEC cytoplasm (Figure 3B, black arrows).

Proplatelet formation appears to start in form of small protrusions of the MZ projecting into the SEC cytoplasm (Figure 3C, red arrowheads). It continues to penetrate more deeply, with a strongly stretched SEC, covering the protrusion to a large extent until MK protrusions transmigrate into the sinusoid lumen (Figure 3C, red arrowheads). Fine filaments anchor the protrusion to the SEC, enabling forward progression at the leading edge of the protrusion (Figure 3C, red arrows).

To study MK-SEC interactions in 3D, we performed SBF-SEM analyses (Figure 4). Representative sections (Figure 4A) and segmentation of the 3D-data set (Figure 4B,C) demonstrate that luminal MK protrusions are still connected to the MK (Figure 4B) through a SEC aperture (Figure 4C, red arrow). Our data reveal the presence of fine extensions of the proplatelets/nascent platelets that connect proplatelet fragments to each other and to the MK cell protrusion (Figure 4B, arrows). Unexpectedly, also the proplatelets developed fine protrusions that were associated with the luminal SEC surface (Figure 4A,B, red arrowheads). The perforation did not occur at sites of intercellular contact of SECs. We thus describe for the first time that nearly mature platelets are not only in contact to each other but also in contact to the opposing SECs.

**Figure 5:**
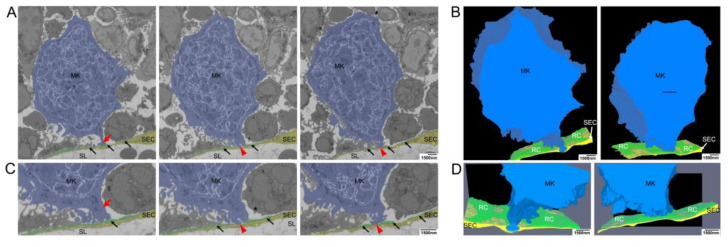
Electron microscopical analysis of local retraction of CAR-cells (reticular cells, RCs) and disintegration of the BL at distinct contact sites of megakaryocyte (MK) protrusions. (**A**) Selected images from single SBF-SEM slices showing a MK (blue) adjacent to the sinusoidal endothelial cells (SECs, yellow). MK protrusions provoke local retraction of RCs (green) at two distinct contact sites where they are associated with the endothelial cell layer. (**B**) Large-volume reconstruction of the SBF-SEM slices presented in (**A**) demonstrating the relationship between a MK protrusion (blue), RCs (black arrows) and SECs (yellow). MK protrusions provoke local retraction of RCs (green) at two distinct sides of SECs contact while these contact sides are still surrounded by RC processes. (**C**) Selected images from single SBF-SEM slices of (**A**) at higher magnification showing a MK protrusion in close association with the blood-BM-barrier. Although the MK protrusion is in close association with this barrier, RCs still cover the endothelial lining to a large extend (red arrow). RCs are retracted locally at two distinct sites where the MK protrusion is associated with the endothelial cell layer (red arrowheads) while the contact sides are still surrounded by reticular cell processes (black arrows). (**D**) Large-volume reconstruction of the SBF-SEM slices presented in (**B**) at higher magnification. SBF-SEM dataset: Reconstruction of a 3.9 μm volume: 132 sections, 10 nm × 10 nm × 30 nm voxel size. BM of the diaphysis region obtained from murine ulnae. Source data are available for this figure. Sinusoidal lumen (SL); Protrusion (P); Marginal zone (MZ).

To further visualize the early phases of MK approach to SECs in three dimensions, additional SBF-SEM images with higher magnification were used for segmentation and 3D reconstruction (Figure 5). MK protrusions initially provoke retraction of reticular CAR-cell processes only at distinct contact sites (Figure 5A,C red arrows, Supplementary Video S1) without any sign of engulfment or penetration of CAR-cells by MK processes. At the lateral border of these contact sites, MK protrusions were still surrounded by RC processes and still attached to the BL of the sinusoid wall (Figure 5A,C black arrows, Supplementary Video S2).

## 4. Discussion

The BM compartment is the main reservoir to provide all blood cells, in order to replenish old and exhausted cells, as well as to produce cells on demand to compensate for blood loss, bleedings or infections. While T- and B-cells mature in thymus or secondary lymphoid organs, extramedullary hematopoiesis in spleen, lung or liver can occur, especially in disease conditions. Nevertheless, the BM provides all blood cell precursors before mature cells are released into the blood stream. The blood-BM-barrier remains enigmatic with respect to its selective transmission of cells: only mature blood cells leave the BM into the peripheral blood, while immature cells remain in the cavity. One exception are hematopoietic stem cells, which can be triggered to leave the BM by applying G-CSF or CXCR4 inhibitors, a mechanism clinically used to mobilize hematopoietic stem cells (HSCs) for human stem cell transplantation. In contrast, none of the circulating mature cells re-enter the BM, except certain immune cells during inflammatory conditions. Again, HSCs can cross the blood-BM-barrier from blood back into the BM in response to HSC transplantation. The molecular mechanisms of HSC homing into the BM remains ill-defined.

CXCL12-abundant reticular cells were shown to be a component of the HSC niche [16] playing an important role in HSC maintenance, as they constitute the major CXCL12- and SCF-producing cells in the BM [17]. Since BM is a likely organ for metastasis of cancer stem cells, CAR-cells are also discussed to act on the survival of these types of stem cells and therefore on cancer relapse, as reviewed in [18].

In a landmark study, Avecilla et al. could demonstrate that TPO-independent thrombopoiesis is mediated by the CXCL12-expressing perivascular niche through relocation of MK progenitors and promotion of maturation [19]. This was shown to be an effective mechanism to reduce chemotherapy-induced thrombocytopenia. CXCR4 antagonists are clinically used to support HSC mobilization [20] and can improve therapy options in malignant conditions with thrombocytopenia [21]. Due to its role in immune cell development, mutations in CXCR4 can lead to lymphomas such as Waldenström’s macroglobulinemia [22] or congenital WHIM-syndrome [23]. These findings imply that an improved understanding of interactions between CAR-cells and the BM niche is clinically highly relevant, in both health and disease conditions.

The endothelial lining of BM sinusoids has been considered fenestrated or even discontinuous [3], despite decade-long knowledge that the endothelium lining is continuous and overall impermeable. This is reflected by the observation that the heavy metal treatment required for TEM analyses did not lead to significant enrichment into the BM cavity, but accumulated at the luminal side of the endothelial lining (Figure S5).

While it is known that MKs are the immediate precursors of platelets in circulation, the visualization of this process has been hampered by the paucity of MKs in the BM and the calcified cortex of bones, which is a major obstacle for microscopic approaches. De Bruyn and co-workers could demonstrate that cytoplasmic MK “bullae” do not only perforate the sinusoidal lining, but also the parasinusoidal reticulum cells [2]. Recent work by Eckly et al. provided elegant experimental evidence that actin-rich podosome-like structures contribute to the MK transendothelial passage through endothelial pores that are newly formed [8]. Brown et al. showed in a series of experiments using reconstructions of serial TEM sections and of large-volume electron tomographic analyses that protruding MKs lose their MZ and have anchoring points at the sinusoid wall during the passage [7]. Both studies provide convincing work on the MK-endothelial interaction. However, none of these considered the effect of the ECM or the RCs that cover the SECs.

Two seminal studies on the migration of blood cells through the sinusoidal wall reported that the BL is often observed between the SECs (regardless of their size) and the adventitial cells [2,24]. Laminin-α5 and collagen types IV and I were unambiguously detected at sites of MK penetration (Figure S2), implying that relevant ECM proteins can be found at these sites. The ECM distribution pattern might contribute to MK polarization when located at the vessel and prevent ectopic platelet release into the BM cavity [16,25]. However, mice lacking both collagen receptors GPVI and integrin-α_2_ did not show ectopic platelet release [26].

Until recently, three-dimensional reconstruction on an ultrastructural level was only possible using serial section transmission electron microscopy (ssTEM) as used in [7]. However, ssTEM is not only time-consuming, but also highly challenging and prone to artifacts as, e.g., section loss, damage, and image distortions. It requires manual alignment of images that could be a further source of artifacts relevant to segmentation of different structures. In addition, ssTEM is often restricted for reconstructions of smaller volumes (as described in [7]). Novel serial scanning electron microscopy methods such as focused ion beam scanning electron microscopy (FIB-SEM as used in [8]) or serial block-face scanning electron microscopy (SBF-SEM) as used in our analysis, overcome these limitations and avoid prohibitive image processing to correct damage and distortion. Both methods allow for collection of data from larger volumes and enable high-resolution imaging and produce reliable serial sections as thin as 25 nm in the case of SBF-SEM, whereas sections used in ssTEM are often restricted to a thickness of 70–80 nm. While FIB-SEM is commonly used for visualizing (sub-)cellular structures in a smaller volume, SFB-SEM has been mainly used for samples that require examination of larger volumes. We therefore chose to use a combination of ssTEM for the initial reconstruction of smaller datasets (data not shown), TEM (Figure S3) and SBF-SEM to investigate how MKs interact with the different components of the blood-BM barrier.

Taken together our data imply that MKs residing at the vascular niche are not in direct contact with SECs that line the luminal surface of the BM sinusoids, but are shielded by CAR cells. In a first step, these CAR cells retract from the site where the transendothelial passage will occur. The active MK will then push with a cytoplasmic protrusion against the SEC and incise into the vessel lumen, taking a thin part of SEC with it, which covers MK protrusions form the luminal side. The MK MZ is maintained at least in the majority of proplatelets and contributes to pushing the MK cytoplasm into the lumen. Inside the vessel, the fragmenting proplatelet will have small cytoplasmic bridges to the luminal surface of SECs before platelets are finally sized and released. These subsequential morphogenetic events of transendothelial passage are summarized in Figure S4.

Taken together, our data reveal in yet unprecedented resolution new details on which morphogenetic events take place at the micro-interface between MKs and SECs in a 3D perspective. We suggest a role for CAR-cells as gatekeepers at this interface, preventing vs. enabling the direct MK-SEC interaction and thus regulating the MK-protrusions into the BM-sinusoid lumen that release platelets into the blood circulation. This will help to further decipher the mechanisms underlying the final steps in thrombopoiesis.

## Data Availability

Not applicable.

## Supplementary Materials

The following are available online at https://www.mdpi.com/2073-4409/10/4/722/s1, Figure S1: Femur tilescan of a CXCL12-dsRed Reporter mouse, Figure S2: Confocal imaging of murine femur bone marrow. Figure S3: TEM images of murine bone marrow sinusoids. Figure S4: Cartoon depicting the transendothelial passage, Figure S5: TEM images of murine bone marrow sinusoids., Supplementary Video S1: MK-BM sinusoid interaction (segmented SBF-SEM data stack), Supplementary Video S2: Higher magnification of the ROI from the MK-BM sinusoid interaction area with endothelial engulfment (segmented SBF-SEM data stack).
