# Supplementary material for: CXCL12-Abundant Reticular (CAR) Cells Direct Megakaryocyte Protrusions across the Bone Marrow Sinusoid Wall
Source: Cells. 2021 Mar 24;10(4):722. doi: 10.3390/cells10040722 (PMC8063926; doi:10.3390/cells10040722)
Supplement: Supplementary file 1 [file cells-10-00722-s001.zip › cells-1138272_supplementary_material.pdf]

CXCL12-abundant reticular (CAR) cells direct megakaryocyte protrusions across the bone marrow sinus wall

Supplemental data

Nicole Wagner[1]*, Kristina Mott[2]*, Berin Upcin[1], David Stegner[2], Harald Schulze[2]*, Süleyman Ergün[1]*

[1]Institute of Anatomy and Cell Biology, Julius-Maximilians-University Würzburg, Germany
[2]Institute of Experimental Biomedicine, University Hospital Würzburg, Germany

*: NW and KM are shared first authors, HS and SE are shared last authors.

Short title: CAR cells direct proplatelet formation

Correspondence:

Prof. Dr. Harald Schulze
University Hospital Würzburg
Institute for Experimental Biomedicine, Chair I
Josef-Schneider-Str. 2/D15
97080 Würzburg, Germany
Phone: +49-931-201-48329
Fax: +49-931-201-648363
harald.schulze@uni-wuerzburg.de

Prof. Dr. Süleyman Ergün
Julius-Maximilians-University Würzburg
Institute of Anatomy and Cell Biology
Koellikerstr. 6
97070 Würzburg, Germany
+49-931-31-82707
+49-931-31-682707
sueleyman.erguen@uni-wuerzburg.de

Supplementary Methods

Electron microscopy

Animals were anesthetized and perfused with 0,9% NaCl + 0.66mL Heparin/100mL (in A. bidest) for 2 minutes followed by 0.15M cacodylate buffer pH 7.4 containing 2.5% glutaraldehyde, 2% formaldehyde (fresh from paraformaldehyde) with 2mM calcium chloride at RT for 5 minutes. Bones (ulnae) were removed and cleaned from muscles, tendons and connective tissue. Bones were cut through the middle of the marrow-containing region with a fresh razor blade. Bone fragments were postfixed in the fixation solution (0.15M cacodylate buffer pH 7.4 containing 2.5% glutaraldehyde, 2% formaldehyde with 2mM calcium chloride) on ice for 2-3 hours. Bone fragments were either further processed for TEM or SBF-SEM as described below.

For transmission electron microscopy (TEM), bone fragments were washed after fixation for 5 x 3 minutes in cold 0.15M cacodylate buffer (50mM cacodylate, 50mM KCl, 2.5mM $MgCl_2$, pH 7.4) on ice. Bone fragments were then decalcified for one week in the decalcification solution (4% paraformaldehyde, 0.85% NaCl, 10% acetic acid; aqueous solution) under stirring. The solution was changed daily. After washing 10 x 5 minutes with ddH_2O, bone fragments were subsequently fixed for 1 hr with 1% osmium tetroxide (buffered with phosphate-buffered saline, PBS, pH 7,2) and washed 5 x 3 minutes with ddH_2O at RT. Bone fragments were dehydrated in an ascending ethanol series using solutions of 30%, 50%, 70% ethanol, 10 minutes each, and incubated for 1 hr with 2% uranylacetate in 70% ethanol. Dehydration was proceeded with incubation of specimens in 70%, 80%, 90%, 96%, 100%, 100% ethanol, 10 minutes each. Bone fragments were incubated two times in propylene oxide (PO) for 30 minutes each before incubation in a mixture of PO and Epon812 (1:1) overnight. The following day, the Epon-PO mixture was substituted with pure Epon812 and bone fragments were incubated for 2 hours in Epon812. Specimens were embedded in Epon812 and kept at 60°C for 48hrs. For ultrathin sections, 70nm thick ultrathin sections were cut with an ultramicrotome (Ultracut E, Reichert Jung, Germany) and collected on copper or nickel grids. Sections were post-stained with 2.5% uranyl acetate and 0.2% lead citrate and finally analyzed with a LEO AB 912 transmission electron microscope (Carl Zeiss Microscopy GmbH, Germany).

For SBF-SEM, bone fragments were prepared using a modification of the NCMIR protocol.[1] Briefly, bone fragments were washed 5 x 3 minutes in cold 0.15M cacodylate buffer (50mM cacodylate, 50mM KCl, 2.5mM $MgCl_2$, 2mM $CaCl_2$ pH 7.4) on ice. Subsequently, bone fragments were incubated on ice in a reduced osmium solution containing 2% osmium tetroxide, 1.5% potassium ferrocyanide, 2 mM $CaCl_2$ in 0.15 mM sodium cacodylate buffer (pH 7.4). Bone fragments were washed with ddH_2O at room temperature (RT) 5 x 5 minutes followed by incubation in 1% thiocarbohydrazide (TCH) solution for 25 minutes at RT. Bone fragments were washed with ddH_2O at RT, 5 x 5 minutes each and incubated in 2% osmium

tetroxide in ddH_20 for 30 minutes at RT. Subsequently, bone fragments were washed 5 x 5 minutes at RT in ddH_2O then incubated in 1% uranyl acetate (aqueous) and stored at 4°C overnight. The next day, bone fragments were washed 3 x 3 minutes in ddH_2O at RT. Prior incubation with lead aspartate solution, bone fragments were washed 2 x 3 minutes in ddH_2O at 60°C and than subjected to *en bloc* Walton's lead aspartate staining[2] and placed in a 60°C oven for 30 minutes. Bone fragments were washed 5 x 5 minutes with ddH_2O at RT. Subsequently, bone fragments were decalcified for one week in the decalcification solution under stirring. The solution was changed daily. Bone fragments were washed 10 x 5 minutes with ddH_2O at RT and dehydrated using ice-cold solutions of freshly prepared 30%, 50%, 70%, 90%, 100%, 100% ethanol (anhydrous), 100% aceton (anhydrous) for 10 minutes each, then placed in anhydrous ice-cold acetone and left at RT for 10 minutes. Bone fragments were placed in 100% aceton at RT for 10 minutes. During this time, Epon812 was prepared. The resin was mixed thoroughly and samples were placed into 25% Epon:acetone for 2 hours, then into 50% Epon:acetone for 2 hours and 75% Epon:acetone for 2 hours. Bone fragments were placed in 100% Epon overnight. The next day, Epon was replaced with fresh Epon for 2 hours and bone fragments were placed in beem capsules and incubated in a 60°C oven for 48 hours for resin polymerization. The resin embedded bone fragments were mounted on aluminium pins (Gatan Inc). The blocks were precision trimmed with a glass knife to expose the bone marrow. Silver paint (Gatan Inc) was used to coat the edges of the tissue block to reduce charging during imaging using BSE mode using the variable pressure mode.

Serial block face imaging

Images were acquired using a scanning electron microscope (Sigma300VP; Carl Zeiss Microscopy GmbH, Germany) equipped with an automated ultramicrotome inside the vacuum chamber (3View; Gatan Inc). The ultramicrotome cut successive sections at a thickness of 30 nm. After each section, the sampleblock-face was scanned. The microscope, the stage and the ultramicrotome were controlled using DigitalMicrograph software GMS3 (Gatan Inc). The SEM was operated in variable pressure mode (VP-Mode) The sample was scanned in VP-Mode with a chamber pressure of 15 Pa and a landing energy of 3,5 kV. Imaging in VP-Mode reduced artifacts arising from sample charging due to high amounts of epon in the bone marrow. The analyzed ROI had a dimension of 7112x6907 pixels with a pixel size of 10 nm.

Supplemental References

1. Deerinck, Thomas J., et al. "NCMIR methods for 3D EM: a new protocol for preparation of biological specimens for serial block face scanning electron microscopy." *Microscopy* 6.8 (2010).

2. Walton, J. U. D. I. E. "Lead asparate, an en bloc contrast stain particularly useful for ultrastructural enzymology." *Journal of Histochemistry & Cytochemistry* 27.10 (1979): 1337-1342.

Supplementary Figures

Supplementary Figure 1

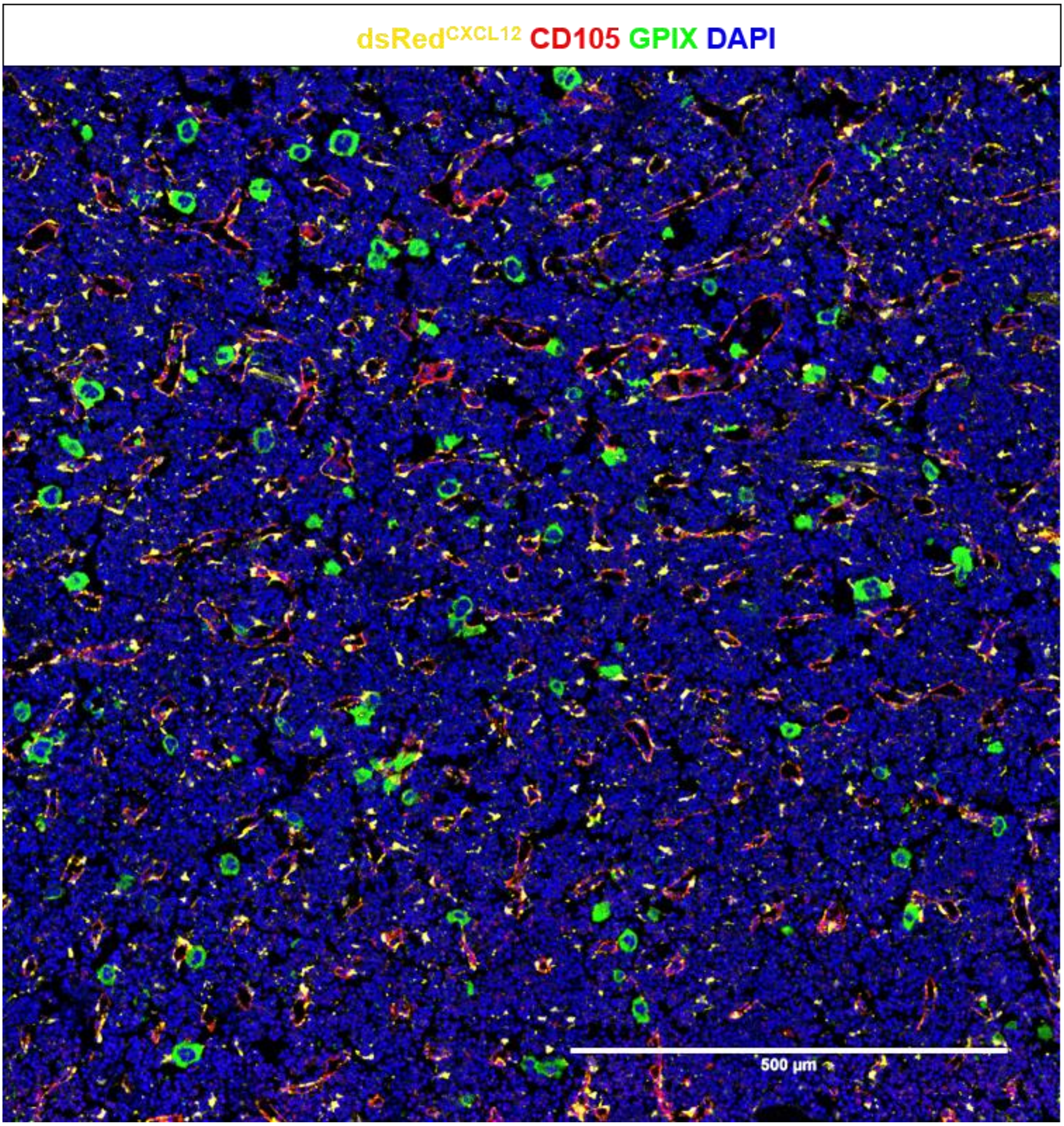

Figure S1: Femur tilescan of a CXCL12-dsRed Reporter mouse. MKs (GPIX, green) and CAR-cells (dsRed^{CXCL12}, yellow) are evenly distributed on the BM. Vessels (CD105) are depicted in red, nuclei (DAPI) are shown in blue. Both MKs in the BM cavity as wells as vessel-residing MKs are in contact with CAR-cells. Scale bar as indicated.

Supplementary Figure 2

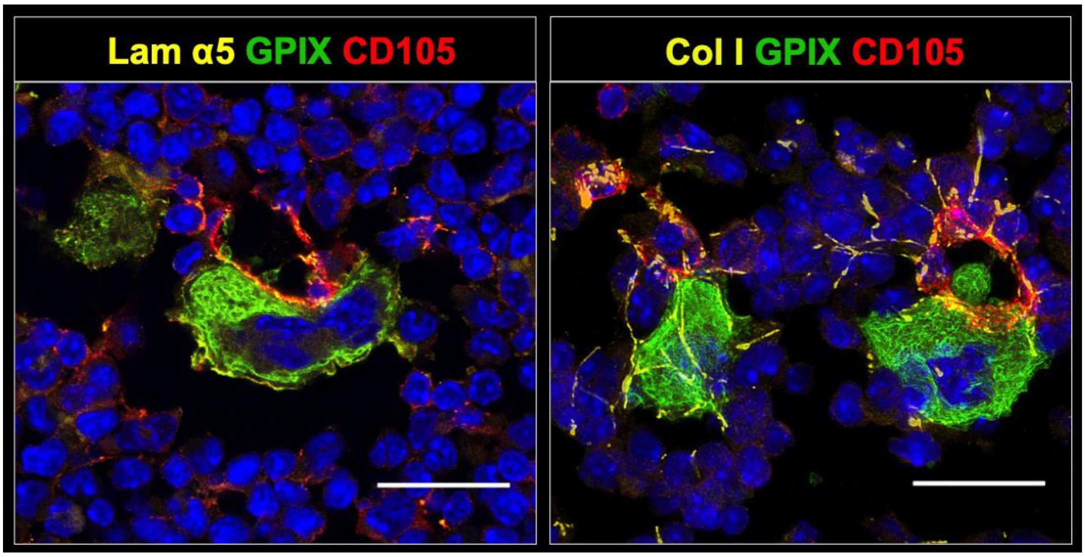

Figure S2: Confocal imaging of murine femur bone marrow. Co-staining of MKs, (GPIX, green) vessels (CD105, red) and laminin-α5 (Lam α5, yellow; left panel) or collagen type I (yellow; right panel). Laminin-α5 can be detected at the sinusoids and is in contact with the extravasating MK. Maximum projection of MKs, vessels and collagen type I co-staining. Collagen I is distributed over the bone marrow cavity and partially co-localizes with CD105-positive sinusoids. Scale bars 20µm.

Supplementary Figure 3

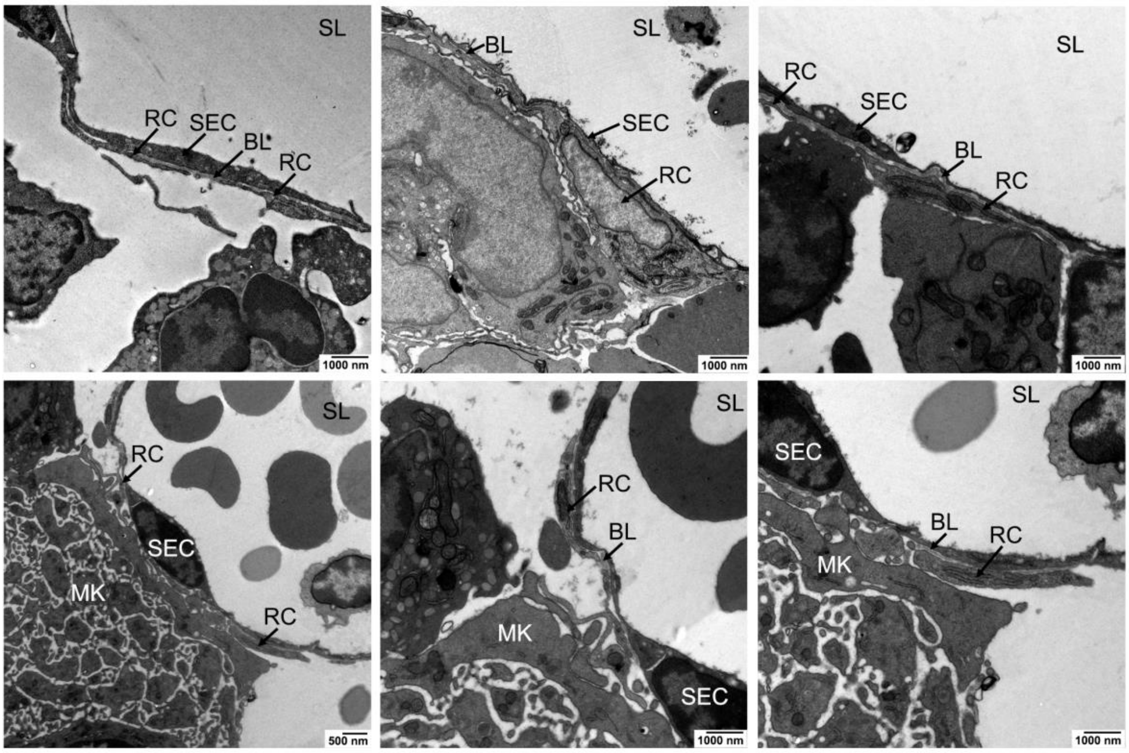

Figure S3: TEM images of murine bone marrow sinusoids. Reticular cells of the BMS forming elongated processes that line the basal site of endothelial cells at sites of non-protrusion (upper row). In most cases of MK approaching to the sinusoids, RC processes were found to be positioned to both sides of the MK process (lower row). RC: Reticular cell, SEC: sinus endothelial cell, BL: basal lamina, SL: sinus lumen. Scale bars as indicated.

Supplementary Figure 4

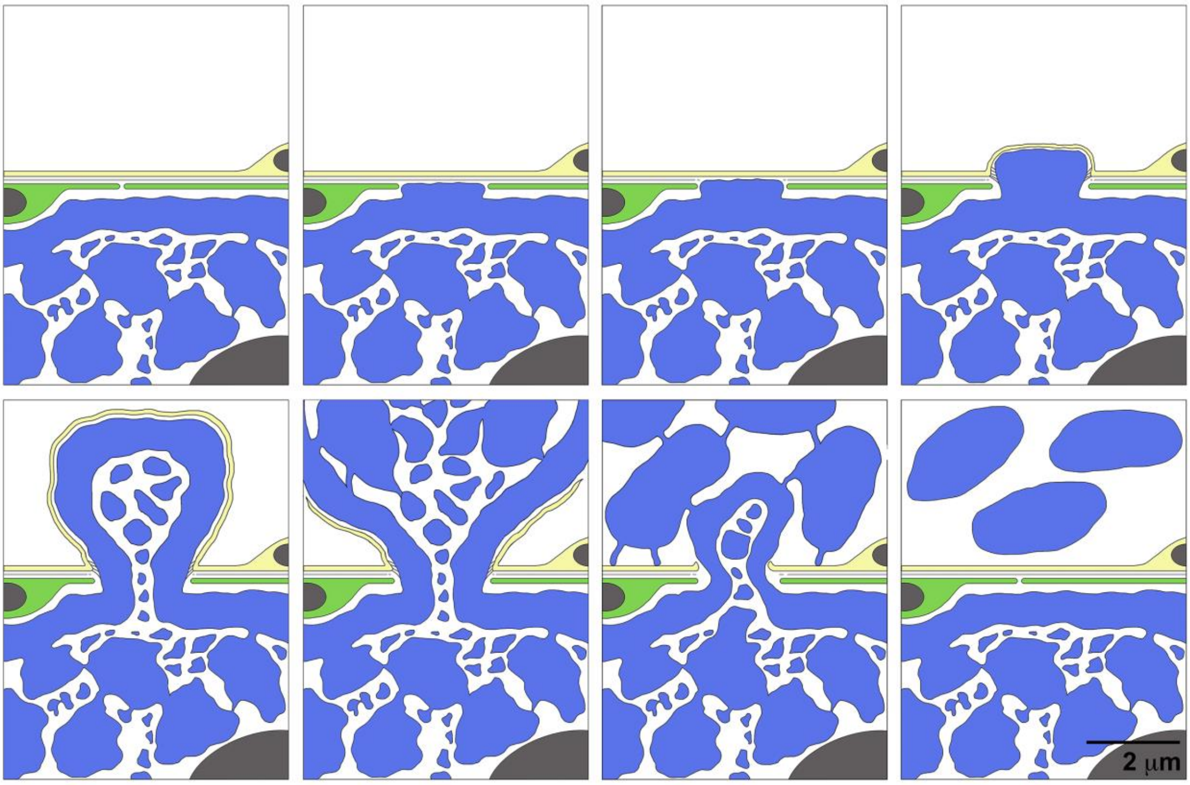

Figure S4: Cartoon depicting the transendothelial passage: First, CXCL12-abundant reticular (CAR) cells (green) retract, before MK protrusions (blue) including the organelle-free zone build an aperture in the endothelial cell (yellow) of the sinus wall. A part of the membrane is pushed out, while the proplatelet protrusion is extravasating into the vessel lumen. While the protrusions further fragment, future proplatelets and platelets are still connected to the luminal side of the endothelial lining. Finally, platelet-sized particles are released.

Supplementary Figure 5

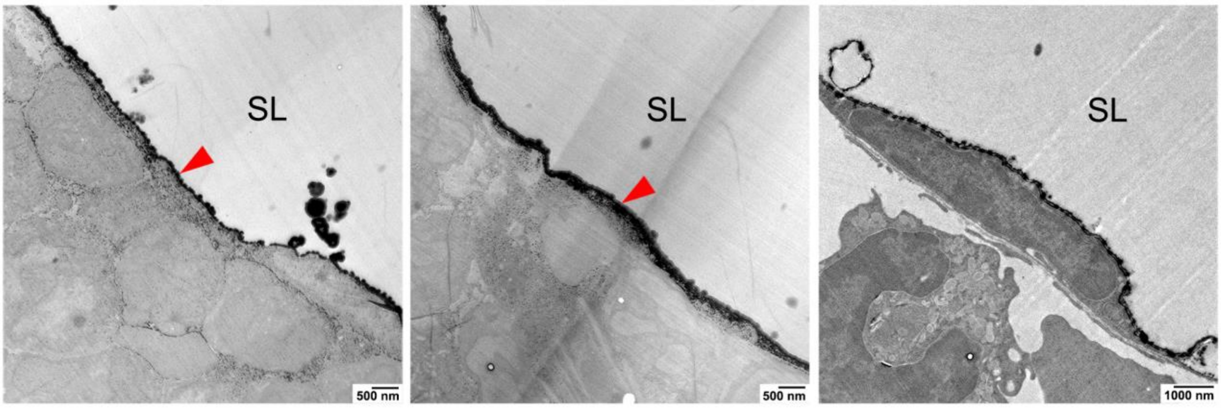

Figure S5: TEM images of murine bone marrow sinusoids. Accumulation of heavy metals at the apical surface of SECs after sample preparation for SBF-SEM (red arrowhead) demonstrating that there is no evidence of any leakage of contrasting agent-containing fixative as it would be expected if the sinusoids were discontinuous or if MK protrusions would open intercellular junctions. SL: sinus lumen. Scale bars as indicated.

Supplementary Videos

Supplementary Video 1. Segmentation of SBF-SEM images. Video of SBF-SEM images shows a MK (blue) approaching the sinusoidal endothelial cells (SECs, yellow). MK protrusions provoke retraction of reticular cells (RC, green) at distinct contact sites.

Supplementary Video 2. Segmentation of SBF-SEM images at higher magnification. A MK (blue) protrusion approaches the sinusoidal endothelial cells (SECs, yellow) and provokes retraction of reticular cells (RC, green) at contact sites.
